# Towards personalised dosimetry in patients with liver malignancy treated with ^90^Y-SIRT using in vivo-driven radiobiological parameters

**DOI:** 10.1186/s40658-022-00479-7

**Published:** 2022-07-30

**Authors:** Yaser H. Gholami, Kathy P. Willowson, Dale L. Bailey

**Affiliations:** 1 Faculty of Medicine and Health, The University of Sydney, Sydney, Australia; 2 Sydney Vital Translational Cancer Research Centre, University of Sydney, Sydney, Australia; 3 Department of Nuclear Medicine, Royal North Shore Hospital, Sydney, Australia

**Keywords:** ^90^Y, Radioembolisation, SIRT, Dose, Response

## Abstract

**Background:**

The prediction of response is one of the major challenges in radiation-based therapies. Although the selection of accurate linear–quadratic model parameters is essential for the estimation of radiation response and treatment outcome, there is a limited knowledge about these radiobiological parameters for liver tumours using radionuclide treatments.

**Methods:**

The “clinical radiobiological” parameters ($T_{{\text{p}}}$, $T_{{\text{k}}}$, $\alpha$, $\alpha {/}\beta$) for twenty-five patients were derived using the generalised linear–quadratic model, the diagnostic ([^18^F] FDG PET/CT) and therapeutic ([^90^Y]-SIR-Spheres PET/CT) images to compute the biological effective dose and tumour control probability (TCP) for each patient.

**Results:**

It was estimated that the values for $\alpha$ and $\alpha {/}\beta$ parameters range in ≈ 0.001–1 Gy^−1^ and ≈ 1–49 Gy, respectively. We have demonstrated that the time factors, $T_{{\text{p}}}$, $T_{{\text{k}}}$ and $T_{{{\text{critic}}}}$ are the key parameters when evaluating liver malignancy lesional response to [^90^Y]SIR-Spheres treatment. Patients with cholangiocarcinoma have been shown to have the longest average $T_{{\text{p}}}$ (≈ 236 ± 67 d), highest TCP (≈ 53 ± 17%) and total liver lesion glycolysis response ($\Delta {\text{TLG}}_{{{\text{liver}}}}$ ≈ 64%), while patients with metastatic colorectal cancer tumours have the shortest average $T_{{\text{p}}}$ (≈ 129 ± 19 d), lowest TCP (≈ 28 ± 13%) and $\Delta {\text{TLG}}_{{{\text{liver}}}}$ ≈ 8%, respectively.

**Conclusions:**

Tumours with shorter $T_{{\text{k}}}$ have shown a shorter $T_{{{\text{critic}}}}$ and thus poorer TCP and $\Delta {\text{TLG}}_{{{\text{liver}}}}$. Therefore, these results suggest for such tumours the [^90^Y]SIR-Spheres will be only effective at higher initial dose rate (e.g.  > 50 Gy/day).

## Background

Selective internal radionuclide therapy (SIRT) with yttrium-90 [^90^Y]-microspheres (SIR-Spheres; Sirtex, Sydney, Australia) (^90^Y-SIRT) has been used as a locoregional therapy for liver metastases of malignancies including neuroendocrine tumours (NETs) and colorectal cancer (CRC) [1, 2], advanced hepatocellular carcinoma (HCC) [3–6] and intrahepatic cholangiocarcinoma (ICC) [7–9]. Although SIRT is a well-established radionuclide therapy (RNT) platform, there are ongoing efforts to further improve treatment planning using personalised dosimetry [10]. The primary principle of radiation therapy is to be able to accurately plan and deliver effective doses to the tumour while minimising the dose to healthy tissues. Knowing the true absorbed dose to tissue compartments is the primary way to safely individualise therapy for maximal response while respecting normal tissue tolerances. Recent progress in positron emission tomography (PET)/computed tomography (CT) imaging has improved the ability to estimate absorbed ^90^Y doses and achieve a more accurate dosimetric approach to the activity calculation in ^90^Y-SIRT [11–14].

Furthermore, one of the major challenges in radiation therapy (both RNT and external beam radiotherapy, EBRT) is to predict the radiobiological response from a particular dose delivered to a tumour [15, 16]. Amongst the established radiobiological models, the linear–quadratic (LQ) model has been best validated by experimental and clinical data and is commonly used to analyse both in vitro and in vivo clinical dose response [17, 18]. In addition to considering the effects of cellular lethal (i.e. DNA double-strand break, DSB) and sub-lethal damage by radiation, the generalised LQ model (GLQ) also includes the effect of the dose rate and the cell proliferation effects [19–21]. Clinically, the GLQ model is increasingly used to predict tumour control probability (TCP) [19–21].

In previous studies [22, 23], we have demonstrated that an in vitro metabolic assay and in vivo metabolic imaging (i.e. Fluorine-18 [^18^F]-fluorodeoxyglucose PET imaging (FDG PET) can be used to derive radiobiological parameters and assess the prognostic factors for radioembolisation of liver metastases from colorectal cancer. Furthermore, our in vitro study demonstrated that dose rate variation can significantly impact the ^90^Y-SIRT dose response. Since the initial dose rate decreases exponentially over time, at a critical time ($T_{{{\text{critic}}}}$) or critical dose rate ($R_{{{\text{critic}}}}$) the DNA damage (the probability of causing DSBs) effectively becomes insignificant [23] due to the rate of DNA repair. Both $T_{{{\text{critic}}}}$ and $R_{{{\text{critic}}}}$ are obtained based on the $\alpha$ parameter (radiosensitivity of the tumour cell), the radionuclide half-life, initial dose rate, and the cell repopulation time ($T_{{\text{p}}} )$ [23]. The other key radiobiological parameter that adversely affects the local tumour control and/or survival is the ‘kick-off’ time ($T_{{\text{k}}}$) [24]. Previous studies have shown that for certain cancer types (particularly highly proliferating cancers with $T_{{\text{p}}}$ ranging from a few days to a few weeks) there exists a short period of time after the start of radiotherapy before the tumour starts to grow more rapidly than prior to irradiation [24–28] and this is referred to as the ‘kick-off time’. Since the dose rate in RNT is mono-or bi-exponentially decreasing and the treatment time is usually long (*e.g.* weeks to months), the $T_{{\text{k}}}$ can significantly impact the biological effective dose (BED) and treatment outcome (e.g. tumour control probability, TCP) [24].

The prediction of response is one of the major challenges in radiation-based therapies. Although the selection of accurate LQ parameters for $T_{{\text{p}}}$, $T_{{\text{k}}}$, $\alpha$, and $\alpha {/}\beta$ is pivotal for a reliable estimate of radiation response and treatment outcome, there is a limited knowledge about these radiobiological parameters for liver tumours [29]. To obtain accurate dosimetry and tumour response prediction for ^90^Y-SIRT, personalised characterisation of the individual patient’s radiobiological parameters is required. Therefore, the aim of this study was to develop a model to fit clinical tumour survival fraction data from treated patients with liver malignancy to derive the radiobiological parameters, evaluate the dosimetry and the treatment outcome specific to each patient.

## Methods

### Overview

The clinical radiobiological parameters (i.e. $T_{{\text{p}}}$, $T_{{\text{k}}}$, $\alpha$, $\alpha {/}\beta$) for twenty-five patients were derived using the diagnostic (FDG PET/CT) and therapeutic (^90^Y-SIRT PET/CT) images to compute the BED map, TCP and FDG PET total liver lesion glycolysis (TLG) for each patient. Furthermore, the relationship between the radiobiological parameters and the calculated dosimetric quantities was investigated.

### Patient characteristics

The data for twenty-five patients with liver malignancy including pancreatic neuroendocrine tumours (PNET), colorectal cancer (CRC), pancreatic ductal adenocarcinoma (PDAC), small bowel neuroendocrine tumours (SBNET), hepatocellular carcinoma (HCC), neuroendocrine Carcinoma (NEC) and other metastatic tumours who were treated with [^90^Y]-SIR-Spheres between July 2019 and April 2021 were used in this study. All patients gave informed consent at the time of the procedure for their clinical and image data to be used for further research, education, training, and audit. For each patient a complete imaging set suitable for lesional analysis was available. Similar to our previous study [22], an individual imaging set consisted of baseline FDG PET/CT (acquired ≤ 28 days prior to radioembolisation), ^90^Y-SIRT PET/CT (acquired within 24 h of radioembolisation), and follow-up FDG PET/CT (acquired ≤ 80 days post-radioembolisation). Treatment time is the time interval between the baseline and follow-up FDG, and each patient’s treatment time is listed in Table 1. All patients underwent pre-treatment interventional arterial mapping and abdominal Technetium‐99 m macroaggregated albumin ([^99m^Tc]MAA) single-photon emission computed tomography SPECT/CT imaging prior to treatment to assess the lung shunt fraction and possible extrahepatic uptake to determine the feasibility, safety, and number of injections required for selective treatments.

**Table 1:** Summary of estimated radiobiological parameters

Cancer type	$T_{{\text{t}}}$ (days)	$T_{{\text{p}}}$ (days)	$T_{{\text{k}}}$ (d)	$\alpha$ (Gy^−1^)	$\alpha {/}\beta$ (Gy)
Breast	49	124	23	0.009	5.00
PNET	47	166	22	0.009	11.2
Adrenocortical carcinoma	49	136	9.0	0.005	4.21
CRC	53	170	26	0.005	49.8
Cholangiocarcinoma	42	200	41	0.015	1.00
CRC	42	108	2.0	0.006	5.83
CRC	49	180	29	0.008	4.00
Rectal adenocarcinoma	46	300	27	0.200	5.91
Cholangiocarcinoma	48	348	48	0.100	1.53
CRC	47	87.0	10	0.010	6.45
Cholangiocarcinoma	47	59.0	1.0	0.001	5.98
Cholangiocarcinoma	48	336	23	0.050	1.00
Sigmoid adenocarcinoma	77	232	27	0.200	6.67
Breast	55	180	55	0.080	1.00
Prostate	56	250	56	0.100	2.00
CRC	56	97.0	50	0.030	4.29
PNET	23	134	49	1.000	2.20
PDAC	49	180	49	0.300	1.00
SBNET	59	300	59	0.800	8.89
Mesothelioma	56	157	39	0.010	8.13
PNET	48	200	48	0.124	12.4
HCC	50	90.0	10	0.003	2.78
Oesophageal	47	200	46	0.050	1.25
SBNET	54	86.0	13	0.022	11.1
NEC	41	87.0	1.0	0.028	1.40

$T_{{\text{t}}}$, treatment time; $T_{{\text{p}}}$, cell repopulation time; $T_{{\text{k}}}$, kick-off time; *PNET* pancreatic neuroendocrine tumours, *CRC* colorectal cancer, *PDAC* pancreatic ductal adenocarcinoma, *SBNET* small bowel neuroendocrine tumours, *HCC* hepatocellular carcinoma, *NEC* neuroendocrine carcinoma.

### Image analysis

All imaging data were acquired using similar scanners and protocols that were used in our previous study [22]. Images were acquired on a Siemens Biograph mCT-S (64) PET/CT system (Knoxville, TN, USA) with 550 picosecond timing resolution time-of-flight (ToF) capabilities, an axial field of view of 21.8 cm and 78 cm crystal ring diameter. Images (with voxel size = 4.072 × 4.072 × 2 mm) were reconstructed using the standard OSEM (with 3 iterations and 21 subsets) reconstruction method in conjunction with ToF modelling and point spread function recovery. Our ‘low-dose protocol’ (i.e. as two 10 min frames over the liver and reconstructed with 3i21s and a 5 mm Gaussian filter) was used to acquire baseline and follow-up PET/CT data. The quantitative liver ^90^Y PET/CT data were reconstructed with 1i21s with no filtering. Siemens’ Intevo-6 or Symbia.T16 were used to acquire the MAA planning SPECT/CT data with low energy parallel hole collimators and standard CT-based attenuation correction were used for reconstruction. To avoid breakdown of the ^99m^Tc-MAA in vivo, in all cases acquisition was performed within 1 h following implantation. The [^90^Y]-microsphere dosimetry navigator software (RapidSphere®) within a commercial platform (Velocity, Varian Medical Systems, Palo Alto, USA) was used to analyse the patient images including the absorbed dose. The absorbed dose was calculated using the local deposition method [30–32].

The ^90^Y-SIRT PET/CT images were first registered to the pre‐ and post‐implantation FDG PET/CT images using the deformable image registration package provided. The Velocity deformable registration algorithm applies multiresolution free‐form deformations and an intensity-based B-spline multipass algorithm to provide a high-level deformable image registration accuracy [32, 33]. The deformable registration provided a one‐to‐one correlation between voxels on different images and time points, allowing for mapping of anatomical data and structure sets from [^90^Y]-microsphere PET/CT, pre-treatment FDG PET/CT to follow‐up FDG PET/CT.

### Dose and BED calculations

#### Dose and survival fraction

The ^90^Y dosimetry navigator was used to calculate the lesion absorbed dose distribution from the ^90^Y PET/CT images. Next, the dose and the normalised standard uptake value (SUV) Volume Histogram of the ^90^Y PET/CT and registered pre- and post-treatment FDG images were computed and imported into MATLAB (R2020a) software. In our previous in vitro study [23], it was demonstrated that the measured radiation survival by metabolic cell assay is comparable to that of clonogenic assays. Additionally, the metabolic assay measures all viable cells thus representing cells from a true tumour population rather than just clonogenic cells [34, 35]. The FDG scan uses a glucose analogue and is the most commonly used PET tracer to assess tumour metabolism. Due to increased glucose metabolism in most types of tumours, the FDG PET is widely used clinically for tumour imaging [36]. The FDG uptake in PET imaging is a measure of the tissue glucose metabolism and is usually high in high-grade tumours (e.g. maximum SUV = 11) and relatively low in low-grade (e.g. maximum SUV = 7) tumours [37]. Additionally, in our previous study [23] we demonstrated that in vivo metabolic imaging such as FDG PET can be used to assess the metabolic dose response as well as prognostic factors for radioembolisation of liver metastases from colorectal cancer. Furthermore, the change in tumour voxel SUV ratio at a specific dose level from a serial FDG PET/CT imaging (i.e. pre- and post-treatment FDG images) can be used to model the tumour voxel dose response [38]. In this study, similarly we have proposed that the ratio of voxel SUV from pre- and post-treatment FDG images should represent the metabolic radiation survival fraction (SF) due to [^90^Y]-microspheres irradiation. Therefore, the ratio of voxel SUV from pre- and post-treatment should represent the metabolic radiation survival fraction (SF) due to [^90^Y]-microspheres irradiation. In addition, to account for dose heterogeneity the SF is calculated based on the dose volume histogram (DVH) by:

1 $${\text{SF}} = \mathop \sum \limits_{i} \frac{{V_{i} }}{{V_{0} }} {\text{SF}}\left( {D_{i} } \right)$$

2 $${\text{SF}}\left( {D_{i} } \right) = \frac{{{\text{SUV}}_{{i,{\text{post}} - {\text{FDG}}}} }}{{{\text{SUV}}_{{i,{\text{pre}} - {\text{FDG}}}} }}$$

where $V_{0}$ is the tumour volume and $V_{i}$ is the sub volume corresponding to ^90^Y dose bin $D_{i}$ on the DVH. Hence using Eqs. 1 and 2, the survival fraction of cancer cells in a tumour volume with an initial volume of $V_{0}$ can be estimated by computing the ratio of voxel SUV from pre- and post-treatment FDG images. Next, the voxel SF (VSF) data was fitted to the GLQ model (Eq. 3, using MATLAB software) to estimate the radiobiological parameters, $T_{{\text{p}}}$, $T_{{\text{k}}}$, $\alpha$, $\alpha {/}\beta$. The GLQ fit is commonly used in the field of radiobiology to derive radiobiological parameters both in vitro and in vivo studies [16].

3 $${\text{SF}} = e^{{ - \left( {\alpha D + G\beta D^{2} + \gamma \left( {T_{{\text{t}}} - T_{{\text{k}}} } \right)} \right)}}$$

4 $${\text{SF}} = e^{{ - \left( {\alpha D + G\beta D^{2} - \frac{{T_{1/2} }}{{T_{{\text{p}}} }} \left( {\frac{\ln \left( 2 \right)}{{\alpha R_{0} T_{{\text{p}}} }}} \right) - \left( {\frac{{\ln \left( 2 \right)T_{{\text{k}}} }}{{T_{{\text{p}}} }}} \right)} \right)}}$$

5 $$G = \frac{2}{{D^{2} }} \mathop \smallint \limits_{ - \infty }^{\infty } \dot{D}\left( t \right){\text{d}}t \mathop \smallint \limits_{ - \infty }^{t} \dot{D}^{\prime } \left( {t^{\prime } } \right) e^{{ - \mu \left( {t - t^{\prime } } \right)}} {\text{d}}t^{\prime }$$

For further personalisation of the GLQ, the treatment time ($T_{{\text{t}}}$) was considered to be equal to the critical time ($T_{{{\text{critic}}}}$) to include the effect of initial dose rate used for each treatment [23]:

6 $$T_{{\text{t}}} = T_{{{\text{critic}}}} = \frac{{T_{1/2} }}{\ln \left( 2 \right)}\ln \left( {\frac{\ln \left( 2 \right)}{{\alpha R_{0} T_{{\text{p}}} }}} \right)$$

where *T*_1/2_ is the ^90^Y half-life (i.e. ≈ 2.7 days), $\alpha$ is the cell radiosensitivity, $R_{0}$ is the initial does rate, and $T_{{\text{p}}}$ is the tumour cell proliferation (or repopulation) time. Hence by replacing the $T_{{\text{t}}}$ with $T_{{{\text{critic}}}}$ and also substituting the tumour cell proliferation (or repopulation) constant, $\gamma = \ln \left( 2 \right)/T_{{\text{p}}}$, in Eq. 4 we can obtain $T_{p}$, $T_{{\text{k}}}$, $\alpha$, $\alpha {/}\beta$ parameters. Furthermore, the GLQ model includes the G-factor (or the Lea–Catcheside factor described by Eq. 5) to account for the kinetics of DNA strand break damage and repair in obtaining the true fraction of surviving cells in an irradiated cell population within the tumour [23]. In Eq. 5, the first integral represents the physical absorbed dose. The integrand of the second integral over $t^{\prime }$ refers to the first DNA single-strand break (SSB) of two SSBs needed to cause lethal DNA double-strand (DSB) damage. Also, the integral over $t$ refers to the second SSB of remaining of two SSBs to cause a DSB. The exponential term reflects the repair and therefore reduction in induction of 2 SSB → DSB process due to decreasing dose rate [23]. Also, $\mu$ (which is $\ln \left( 2 \right)/T_{{{\text{rep}}}}$) is the DNA repair time constant ($T_{{{\text{rep}}}}$ is the DNA repair half-life which is ≈ 1.5 hr [23]).

The $R_{{{\text{critic}}}}$ was also calculated by Eq. 7 for further assessment of the treatment outcome.

7 $$R_{{{\text{critic}}}} = \frac{\ln \left( 2 \right)}{{\alpha T_{{\text{p}}} }}$$

Both $T_{{{\text{critic}}}}$ and $R_{{{\text{critic}}}}$ were calculated theoretically using Eqs. 6 and 7 for a range of $\alpha , T_{{\text{p}}}$ and $R_{0}$ shown in Fig. 5a–b and using the clinically driven radiobiological parameters for comparison. Furthermore, to show the impact of the proliferation acceleration on dose rate efficiency during the treatment time, the $R_{{{\text{critic}} \left( k \right)}}$ was also calculated for when the $T_{{\text{p}}}$ ≈ $T_{{\text{k}}}$ (this can represent the highest acceleration in tumour proliferation rate).

#### BED calculation

The computed lesion dose for each patient was used as an input to generate the lesion BED map. The BED calculations were performed considering the following methods:

Method1. Due to low dose rate and relatively long treatment time (^90^Y half-life ≈ 2.7 days), repair of sub-lethal damage might take place during the treatment duration. Additionally, highly proliferating cancers can have short $T_{{\text{k}}}$ (relative to the treatment time) which could impact the survival fraction and TCP. Therefore, treatment with an exponentially decaying source (integrated to fixed treatment time, *T*) and considering the above parameters can be computed as (Method 1):

8 $${\text{BED}}_{\exp } = D \times {\text{RE}} - \frac{{\ln \left( 2 \right)\left( {T - T_{{\text{k}}} } \right)}}{{\alpha T_{{\text{p}}} }}$$

9 $${\text{RE}} = 1 + \left( {\frac{{2R_{0} \lambda }}{\mu - \lambda }} \right)\left( {\frac{\beta }{\alpha }} \right)\left( {\frac{{\frac{1}{2\lambda }\left( {1 - e^{ - 2\lambda T} } \right) - \frac{1}{\mu + \lambda }\left( {1 - e^{{ - \left( {\mu + \lambda } \right)T}} } \right)}}{{1 - e^{ - \lambda T} }}} \right)$$

10 $$R_{0} = \frac{\lambda D}{{1 - e^{ - \lambda T} }}$$

Method 2. This method is the simplified version of Method 1 where treatment time is integrated to $T \to \infty$. This is the standard BED formulation [39] in the radionuclide therapy dosimetry to calculate the BED:

11 $${\text{BED}}_{\infty } = D.{\text{RE}}_{\infty }$$

12 $${\text{RE}}_{\infty } = 1 + { }\left( {\frac{{R_{0} }}{\mu + \lambda }} \right)\left( {\frac{\beta }{\alpha }} \right)$$

#### TCP calculations

For each tumour with *N* voxels, the voxel TCP was calculated first and then the overall expected TCP of tumour was obtained as the product of the expected voxel TCP [40, 41]:

13 $${\text{TCP}}_{{{\text{voxel}}}} = e^{{ - D_{{\text{c}}} V \cdot {\text{VSF}}}}$$

14 $${\text{TCP}} = \mathop \prod \limits_{i = 1}^{N} \left[ {{\text{TCP}}_{{{\text{voxel}}}} } \right]^{\frac{1}{N}}$$

where $D_{{\text{c}}}$ (i.e. $1 \times 10^{6}$) and $V$ are the density of clonogens per cm^3^ and the voxel volume respectively.

#### TLG calculations

Finally, the ${\text{TLG}}_{{{\text{liver}}}}$ of each liver lesion was calculated by multiplying the metabolic tumour volume of that lesion with its corresponding mean SUV and the $\Delta {\text{TLG}}_{{{\text{liver}}}}$ was calculated in percentage as [22]:

15 $$\Delta {\text{TLG}}_{{{\text{liver}}}} \left( \% \right) = \frac{{{\text{TLG}}_{{{\text{pre}}}} - {\text{TLG}}_{{{\text{post}}}} }}{{{\text{TLG}}_{{{\text{pre}}}} }} \times 100$$

where ${\text{TLG}}_{{{\text{pre}}}}$ and ${\text{TLG}}_{{{\text{post}}}}$ are the ${\text{TLG}}_{{{\text{liver}}}}$ values based on pre- and post-treatment FDG PET images.

## Results

A summary of the derived radiobiological parameters and dosimetry is presented in Tables 1, 2 and 3. The average $T_{{\text{p}}}$, $T_{{\text{k}}}$, $\alpha$, $\alpha {/}\beta$ parameters were ≈ 176 ± 16.3 days, 30.6 ± 3.8 days, 0.10 ± 0.05 Gy^−1^ and 7 ± 2 Gy, respectively. The mean dose, ${\text{BED}}_{\infty }$_,_ ${\text{BED}}_{{{\text{exp}}}}$, TCP and $\Delta {\text{TLG}}_{{{\text{liver}}}}$ were ≈ 63.6 ± 14.2 Gy, 97.8 ± 23.6 Gy, 61.6 ± 14.2 Gy, 43.5% ± 5.6% and 45.2% ± 9.0%, respectively. For all the patients, the treatment time $T_{{\text{t}}}$ is greater than or equal to $T_{{\text{k}}}$. Therefore, although there is a variation in the $T_{{\text{t}}}$, this does not influence the derivation of the other parameters [23].

**Table 2:** A list of estimated critical dose rate, $R_{{{\text{critic}}}}$ and time, $T_{{{\text{critic}}}}$

Cancer type	$R_{{{\text{critic}}}}$(Gy/days)	$R_{{{\text{critic}}\left( k \right)}}$(Gy/days)	$R_{0}$(Gy/days)	$T_{{{\text{critic}}}}$(days)
Breast	0.620	3.32	12.3	12
PNET	0.465	3.45	13.1	13
Adrenocortical carcinoma	1.0	16.3	13.5	10
CRC	0.814	5.24	32.8	14
Cholangiocarcinoma	0.231	1.13	7.28	13
CRC	1.0	56.1	8.62	8
CRC	0.481	3.01	11.0	12
Rectal adenocarcinoma	0.012	0.13	3.18	22
Cholangiocarcinoma	0.02	0.14	8.82	23
CRC	0.777	6.76	8.59	9
Cholangiocarcinoma	11.7	693	70.5	7
Cholangiocarcinoma	0.041	0.60	10.2	21
Sigmoid adenocarcinoma	0.015	0.13	13.0	26
Breast	0.048	0.16	11.8	21
Prostate	0.028	0.12	8.65	22
mCRC	0.238	0.46	7.65	13
PNET	5.00 × 10^−3^	0.01	11.0	30
PDAC	0.013	0.05	2.29	20
SBNET	0.003	0.02	59.8	38
Mesothelioma	0.442	1.76	15.5	14
PNET	0.028	0.12	25.4	26
HCC	3.070	26.5	20.9	7
Oesophageal	0.069	0.30	4.58	16
SBNET	0.362	2.36	12.9	14
NEC	0.284	22.4	4.44	11

$R_{{{\text{critic}}}}$, critical dose rate; $R_{{{\text{critic}}\left( k \right)}}$, critical dose rate considering the $T_{{\text{k}}}$; $R_{0}$, initial dose rate; $T_{{{\text{critic}}}}$, critical time.

**Table 3:** Summary of computed dosimetry

Cancer type	Dose (Gy)	BED $\infty$ (Gy)	BED_exp_ (Gy)	TCP (%)	$\Delta {\text{TLG}}_{{{\text{liver}}}}$(%)
Breast	47.7	58.0	41.5	41.7	43.6
PNET	51.4	56.8	45.3	44.2	48.8
Adrenocortical carcinoma	52.7	67.7	26.2	17.6	23.7
CRC	127	170	90.1	65.6	75.5
Cholangiocarcinoma	28.1	46.2	46.1	50.6	56.9
CRC	33.3	37.6	6.03	0.12	− 113
CRC	42.6	53.0	43.3	42.8	47.7
Rectal adenocarcinoma	12.3	12.9	12.6	20.4	25.8
Cholangiocarcinoma	34.0	51.3	51.3	82.5	98.6
CRC	33.2	37.0	8.10	0.10	− 9.73
Cholangiocarcinoma	272	554	11.8	7.31	16.0
Cholangiocarcinoma	39.3	74.6	73.6	71.9	82.6
Sigmoid adenocarcinoma	53.0	62.7	61.9	54.6	70.3
Breast	45.6	93.0	93.0	79.1	89.0
Prostate	62.6	107	107	82.0	96.0
mCRC	29.5	34.2	32.7	32.1	37.5
PNET	42.2	60.9	60.7	50.8	57.0
PDAC	8.84	47.8	47.8	45.0	58.3
SBNET	231	368	368	81.3	85.1
Mesothelioma	59.9	62.5	69.9	60.6	65.7
PNET	97.8	116	116	72.8	80.3
HCC	80.6	134	12.5	0.27	0.15
Oesophageal	37.7	63.6	63.5	51.7	62.0
SBNET	50.0	55.1	40.3	31.5	35.0
NEC	17.1	21.9	10.5	0.32	− 2.29

$\Delta {\text{TLG}}_{{{\text{liver}}}}$, the total liver lesion glycolysis.

Data presented in Table 4 are grouped by cancer type. Patients with breast and cholangiocarcinoma metastatic liver lesions have shown the highest TCP (i.e. 60% and 53%, respectively) and $\Delta {\text{TLG}}_{{{\text{liver}}}}$ (66% and 63%, respectively). Furthermore, these patients have the longest repopulation time (mean $T_{{\text{p}}}$ ≈ 235 ± 67.2 days). Also, patients with metastatic CRC (mCRC, with shortest mean $T_{{\text{p}}}$≈ 128 ± 19.4 days) have shown the poorest TCP and $\Delta {\text{TLG}}_{{{\text{liver}}}}$, 28.2% and 7.7%, respectively. Additionally, it was estimated that patients with mCRC liver lesions have the smallest $\alpha$ (i.e. 0.0100 ± 0.005) with shortest $T_{{\text{k}}}$ (23.5 ± 8.3 days) and $T_{{{\text{critic}}}}$ (i.e. ≈11.4 ± 3.8 days). Patients with CRC have shown the largest variation in TCP (i.e.  = 28 ± 12.7) with COV ≈ 26%. Consistently their derived $\alpha$ (i.e. 0.01 ± 12.7 with COV≈ 50%) and $\alpha {/}\beta$ (i.e. 14.1 ± 8.9 with COV ≈ 50%) parameters have the highest variation amongst all patients. Furthermore, these results show a variation (i.e. COV ≈ 77%) for the derived $\alpha$ parameter across all cancer types.

**Table 4:** Summary of the mean radiobiological parameters for patients with specific cancer type

Cancer type	$T_{{\text{t}}}$ (days)	$T_{p}$ (days)	$T_{{\text{k}}}$ (days)	$\alpha$ (Gy^−1^)	$\alpha {/}\beta$ (Gy)	BED (Gy)	TCP (%)	$\Delta {\text{TLG}}_{{{\text{liver}}}}$(%)
Breast	52.0 ± 3.0 COV ≈ 6%	152 ± 28 COV ≈ 18%	39.1 ± 15.9 COV ≈ 41%	0.045 ± 0.036 COV ≈ 80%	3.0 ± 2.0 COV ≈ 67%	67.2 ± 25.8 COV ≈ 38%	60.4 ± 18.7 COV ≈ 31%	66.3 ± 22.3 COV ≈ 34%
NET	45.3 ± 5.1 COV ≈ 11%	162 ± 33 COV ≈ 20%	32.1 ± 9.5 COV ≈ 30%	0.33 ± 0.18 COV ≈ 55%	7.87 ± 2.0 COV ≈ 25%	106.7 ± 54.1 COV ≈ 51%	46.8 ± 12.0 COV ≈ 26%	50.7 ± 13.1 COV ≈ 26%
CRC	49.4 ± 2.4 COV ≈ 5%	129 ± 19 COV ≈15%	23.5 ± 8.3 COV ≈ 35%	0.010 ± 0.005 COV ≈ 50%	14.1 ± 8.9 COV ≈ 63%	36.0 ± 15.3 COV ≈ 43%	28.2 ± 12.7 COV ≈ 45%	7.7 ± 33.1 COV ≈ 430%
Cholangiocarcinoma	46.3 ± 1.4 COV ≈ 3%	236 ± 67.2 COV ≈ 28%	28.3 ± 10.5 COV ≈ 37%	0.040 ± 0.022 COV ≈ 55%	2.38 ± 1.21 COV ≈ 51%	45.7 ± 12.8 COV ≈ 28%	53.1 ± 16.6 COV ≈ 31%	63.5 ± 18.0 COV ≈ 28%
Others	53.8 ± 3.6 COV ≈ 7%	174 ± 24 COV ≈ 14	32.2 ± 6.2 COV ≈ 19%	0.100 ± 0.040 COV ≈ 40%	4.28 ± 0.94 COV ≈ 22%	40.9 ± 11.5 COV ≈ 28%	35.3 ± 9.5 COV ≈ 27%	42.9 ± 11.0 COV ≈ 26%

*COV* coefficient of variance

**Fig. 1:**
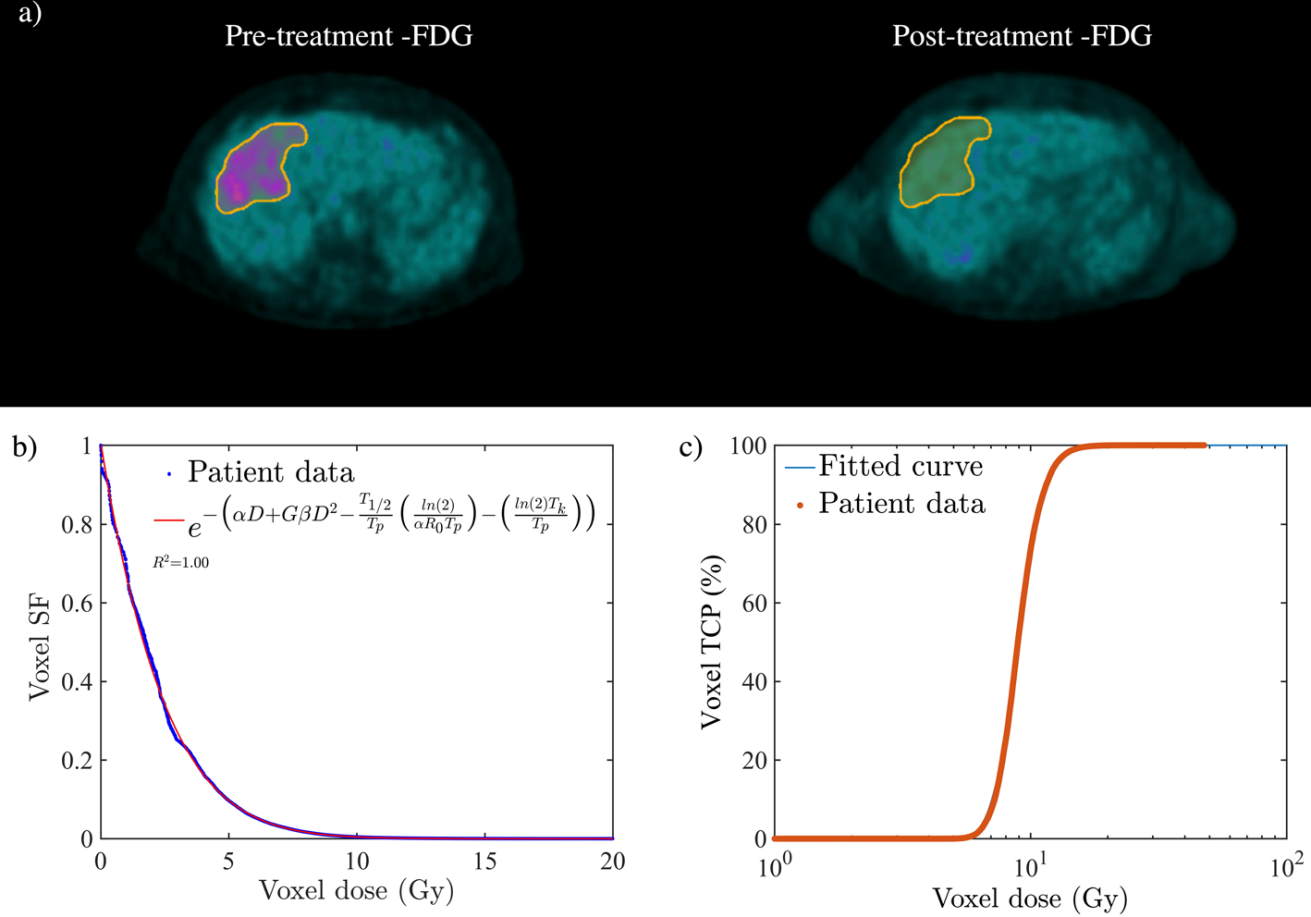
Complete metabolic response (CMR) for a patient with cholangiocarcinoma liver malignancy. Deformable PET images and corresponding calculated voxel survival fraction (VSF) and tumour control probability (TCP). **a** The deformably registered pre‐and post-treatment [^18^F] FDG PET images. The registration provided a one‐to‐one correlation between voxels on pre‐and post-treatment [^18^F] FDG PET images. **b**, **c** The estimated voxel SF and TCP. The survival fraction and TCP were calculated based on the ^90^Y absorbed dose and SUV volume histograms. The radiobiological parameters were derived from the generalised linear–quadratic (GLQ) fit (see Table 1)

**Fig. 2:**
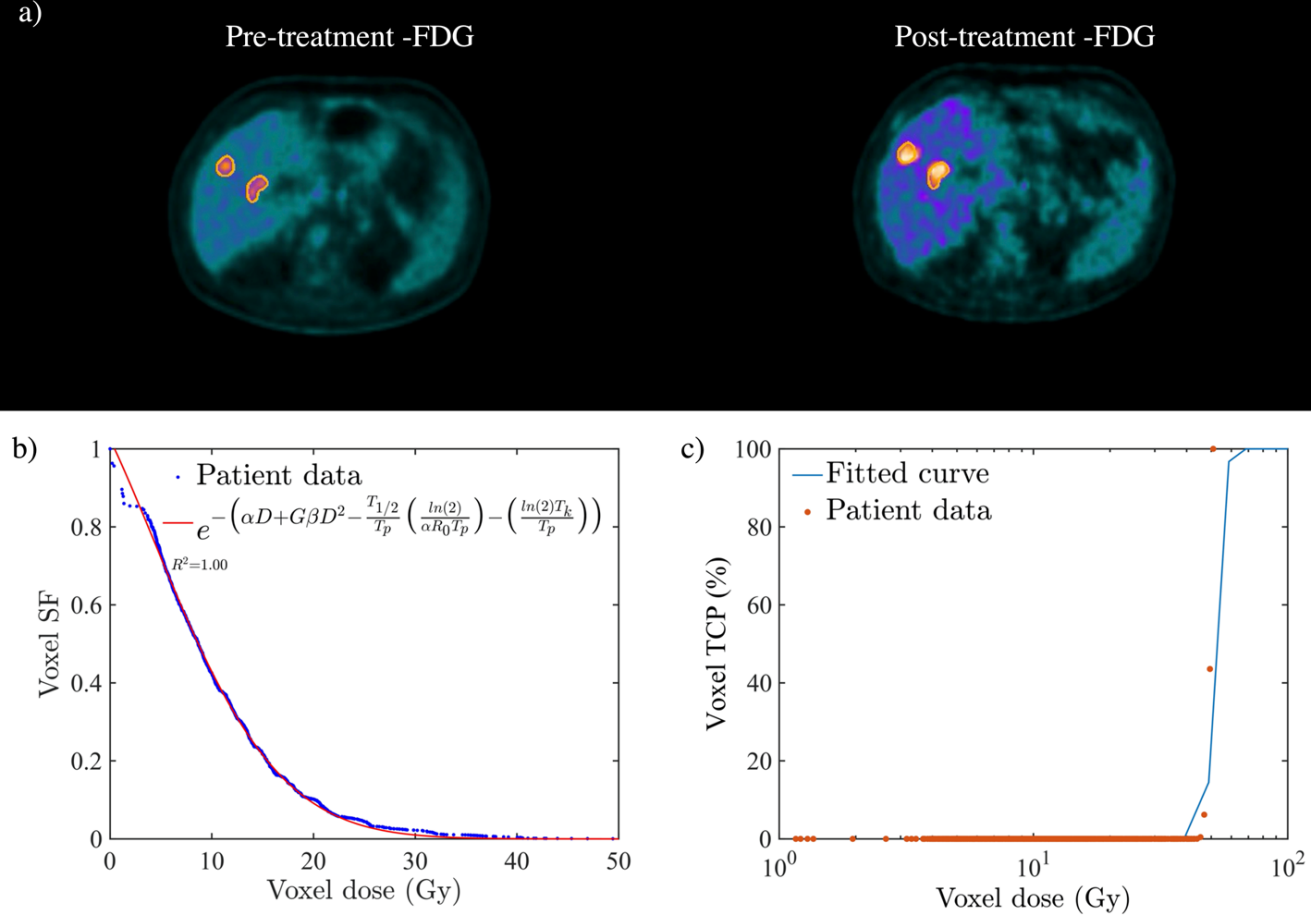
Progressive metabolic disease (PMD) for a patient with metastatic CRC liver malignancy. Deformable PET images and corresponding calculated voxel SF and TCP. **a** The deformably registered pre- and post-treatment [^18^F] FDG PET images. **b**, **c** The estimated voxel SF and TCP. The survival fraction and TCP were calculated based on the ^90^Y absorbed dose and SUV volume histograms. The radiobiological parameters were derived from the GLQ fit (see Table 1)

Figure 1a–c shows the registered pre- and post-treatment FDG PET/CT images and the corresponding [^90^Y]-microspheres VSF and TCP for a cholangiocarcinoma lesion case with complete metabolic response (CMR: 100% reduction in ${\text{TLG}}_{{{\text{liver}}}}$, i.e. lesions not visible above background at the time of follow-up). From the GLQ fit (the blue curve, *R*^2^ ≈ 1), the following average radiobiological parameters were derived, $T_{{\text{p}}}$≈ 348 days, $T_{{\text{k}}}$ ≈ 48 days, $\alpha$ ≈ 0.1 Gy^−1^, $\alpha {/}\beta$ ≈ 1.5 Gy, respectively. Furthermore, the calculated ${\text{BED}}_{\infty }$, ${\text{BED}}_{exp}$, $\Delta {\text{TLG}}_{{{\text{liver}}}}$ and TCP for this case were, ≈ 51.3 Gy, 51.2 Gy, 99% and 83%, respectively. Figure 2a–c also shows the pre- and post-treatment FDG PET/CT images for a patient with mCRC liver lesion with progressive metabolic disease (PMD: more than a 50% increase in ${\text{TLG}}_{{{\text{liver}}}}$, $\Delta {\text{TLG}}_{{{\text{liver}}}}$ > − 50%). In comparison to the previous case, the GLQ fit (the blue curve, *R*^2^ ≈ 1) for this case has shown a much shorter repopulation and kick-off time (i.e. $T_{{\text{p}}}$≈ 108 days, $T_{{\text{k}}}$≈ 2 days, respectively) and thus indicating a more proliferating tumour. The ${\text{BED}}_{\infty }$, ${\text{BED}}_{{{\text{exp}}}}$, TCP and $\Delta {\text{TLG}}_{{{\text{liver}}}}$ for this case were calculated to be ≈ 37.2 Gy, 6.0 Gy, 0.12% and − 112.7%, respectively.

**Fig. 3:**
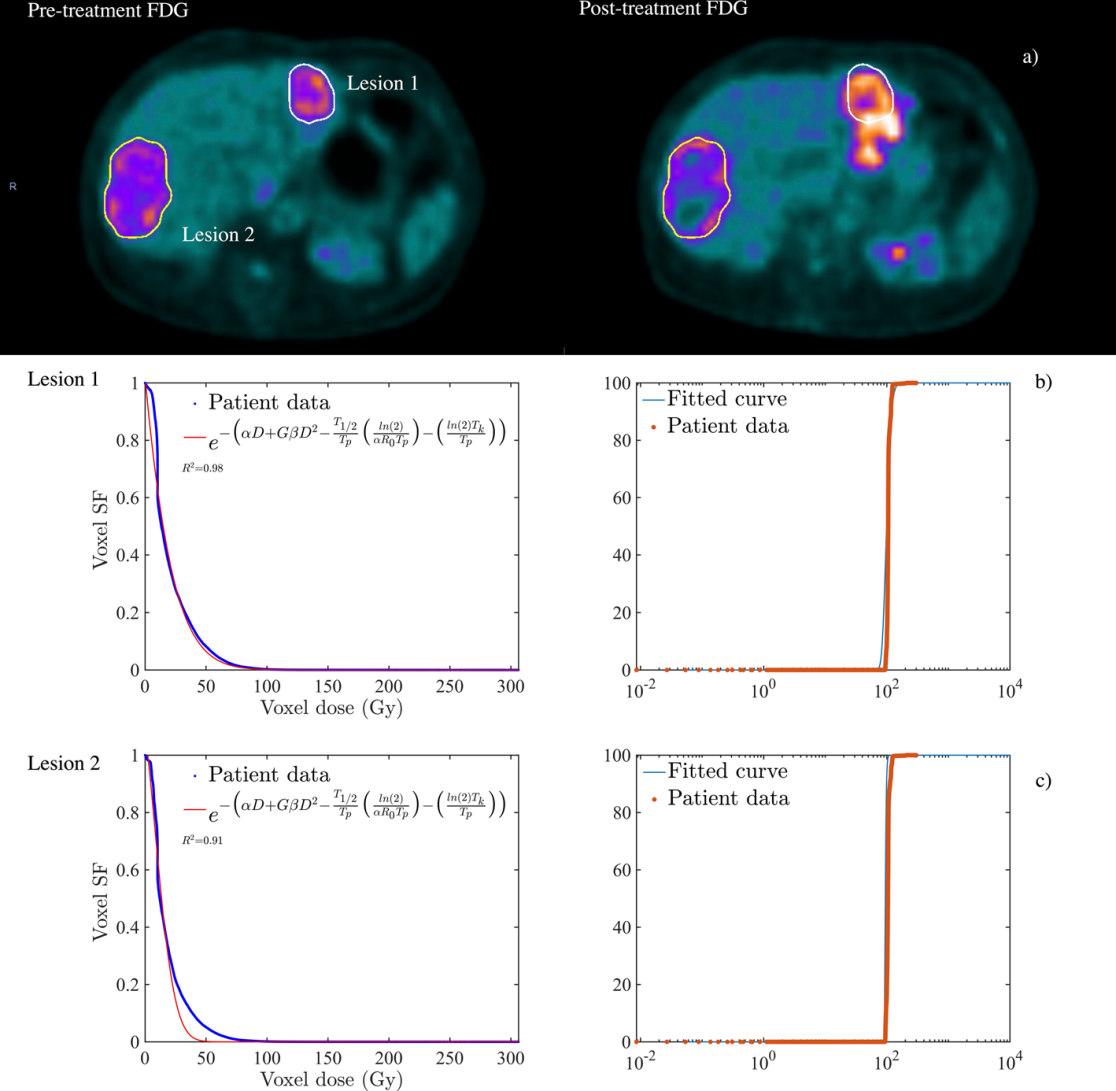
Tumour cell heterogeneity within the metastatic CRC liver malignancy. **a** The deformably registered pre- and post-treatment [^18^F] FDG PET images with two lesions on the right and left lobes are shown with the white contours. **b**, **c** The estimated voxel SF and TCP for both lesions. The survival fraction and TCP were calculated based on the ^90^Y absorbed dose and SUV volume histograms. The radiobiological parameters were derived from the GLQ fit for each individual lesion

**Fig. 4:**
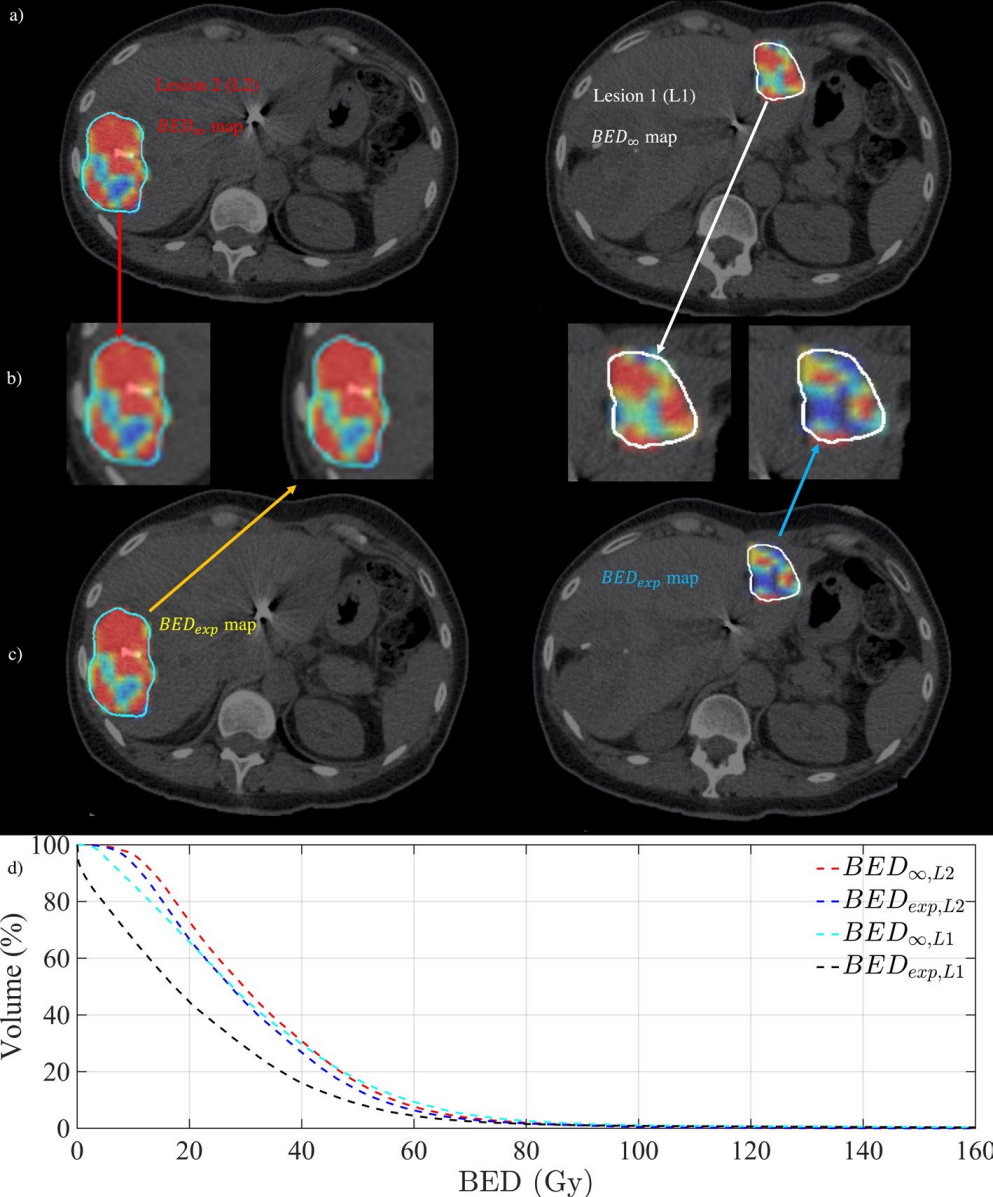
The BED map for lesions shown in Fig. 3. **a**–**c** The ${\text{BED}}_{\infty }$ and ${\text{BED}}_{exp}$ maps for lesion 1 (*L*1) and lesion 2 (*L*2) with the zoom-in of the lesion BED maps. **d** The ${\text{BED}}_{\infty }$ and ${\text{BED}}_{{{\text{exp}}}}$ volume histograms for both lesions

The effect of tumour cell heterogeneity on the voxel SF and TCP is shown in Fig. 3a–b. Figure 3a demonstrates the pre-treatment [^18^F] FDG PET scan for a patient with two metastatic CRC liver lesions (*L*1 and *L*2) in the left and right lobes, respectively. Both lesions (with volumes *L*1 ≈ 79 mL and *L*2 ≈ 105 mL) have received an average ^90^Y dose of ≈ 30 Gy and the voxel SF with the GLQ fit is demonstrated in Fig. 3b. Although, the derived $\alpha$ parameter for both lesions ≈ 0.03 Gy^−1^, *L*1 was shown to have a shorter $T_{{\text{p}}}$ and $T_{{\text{k}}}$ (≈ 92 and 13 days, respectively) in comparison to *L*2 (≈ 179 and 36 days). Moreover, Fig. 3c shows the calculated TCP (≈ 21%) for *L*2 was ≈ 43% higher than the *L*1 TCP (≈ 12%). The TCP results were consistent with the $\Delta {\text{TLG}}_{{{\text{liver}}}}$ (*L*1 ≈ 14% and *L*2 ≈ 22%) and ${\text{BED}}_{{{\text{exp}}}}$ calculations which are shown in Fig. 4a–c.

Figure 4a–c demonstrates the ${\text{BED}}_{\infty }$ and ${\text{BED}}_{{{\text{exp}}}}$ maps for both *L*1 and *L*2 with their corresponding BED volume histograms shown in Fig. 4d, respectively. While *L*2 has a similar ${\text{BED}}_{\infty }$ and ${\text{BED}}_{{{\text{exp}}}}$ value (≈ 31 and 33 Gy, respectively), the *L*1 ${\text{BED}}_{\infty }$(≈ 32 Gy) was 30% higher than ${\text{BED}}_{{{\text{exp}}}}$ (≈ 22 Gy).

**Fig. 5:**
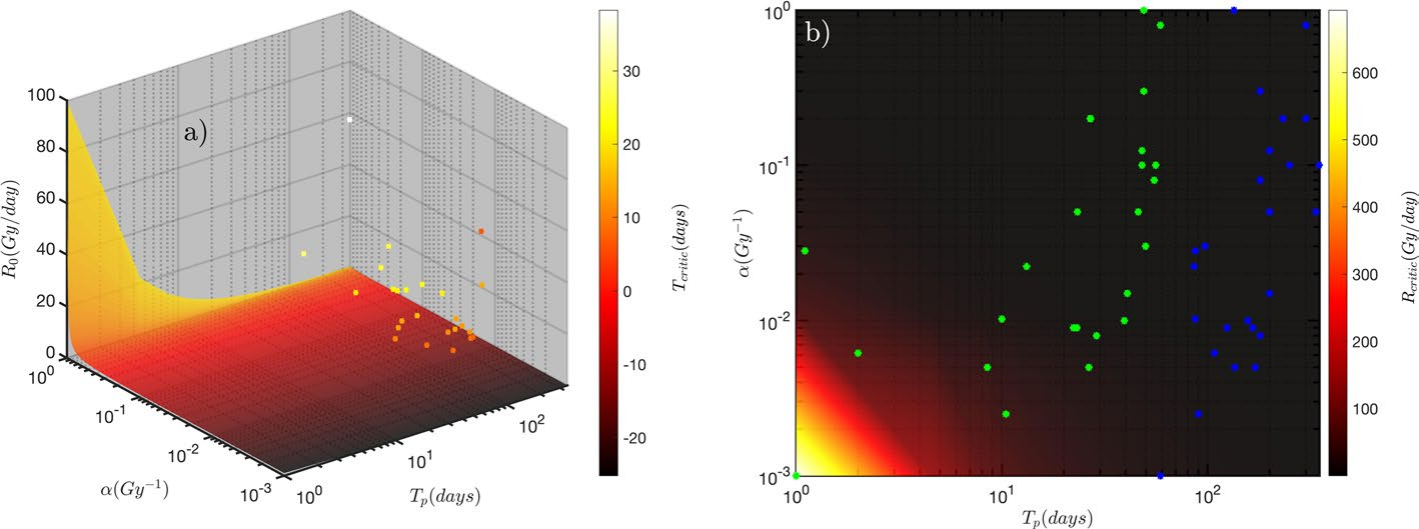
The estimated critical time ($T_{{{\text{critic}}}}$) and dose rate ($R_{{{\text{critic}}}}$). **a** The four-dimensional (4D) theoretical $T_{{{\text{critic}}}}$ spectrum and the estimated clinical $T_{{{\text{critic}}}}$ (dot points) using the radiobiological parameters derived from patient data. **b** The theoretical $R_{{{\text{critic}}}}$ spectrum and the estimated clinical $R_{{{\text{critic}}}}$ and $R_{{{\text{critic}}\left( k \right)}}$ (blue and green dot points, respectively) using the radiobiological parameters derived from patient data (see Table 1). The clinical $R_{{{\text{critic}}}}$ and $R_{{{\text{critic}}\;\left( k \right)}}$ were estimated using the derived repopulation $T_{{\text{p}}}$ and for when $T_{{\text{p}}} = T_{{\text{k}}}$, respectively. The $T_{{{\text{critic}}}}$ and $R_{{{\text{critic}}}}$ spectra are predicted by Eqs. 6 and 7 for a range of $\alpha$, $T_{{\text{p}}}$ and initial dose rate, $R_{0}$ parameters

Figure 5a demonstrates a 4D plot of the theoretical $T_{{{\text{critic}}}}$ spectrum calculated for a range of $\alpha$ (10^–3^–1 Gy^−1^), $T_{p}$(1–350 days) and $R_{0}$ (≈ 1 μGy/day–100 Gy/day). ^90^Y requires approximately 27 days (e.g. 10 half-lives, ^90^Y half-life is ≈ 2.7 days) to deliver more than 98% of a targeted dose. Therefore, for ^90^Y-SIRT, a treatment with a $T_{{{\text{critic}}}}$ less than 27 days will be biologically less effective [23]. The black-red shaded area of the spectrum represents the most inefficient treatment (for low dose rates ≈ 1 μGy/day–1 Gy/day) cases for different combination of $\alpha$ and $T_{p}$ parameters and thus resulting in the shortest critical time ranging between ≈ − 25 and 0 days (the negative $T_{{{\text{critic}}}}$ means no therapeutic effect). Moreover, the spectrum shows that as the initial dose rate increases, the critical times also is prolonged (the yellow-white shaded area) and therefore suggesting a better therapeutic effect could be achieved at higher dose rates. However, the spectrum also shows that for slow proliferating (i.e. longer $T_{{\text{p}}}$, ≈ > 100 days) and radiosensitive lesions (*e.g.* larger $\alpha$ parameters), even at lower initial dose rates (*e.g.* 1 Gy/day), the $T_{{{\text{critic}}}}$ could be as high as ≈ 20 days. Furthermore, the estimated clinical critical times have shown a similar trend to the theoretically calculated $T_{{{\text{critic}}}}$ spectrum. Patients with slow proliferating and radiosensitive lesions have shown to have higher $T_{{{\text{critic}}}}$ compared to other patients.

Furthermore, Fig. 5b demonstrates a 3D plot of the $R_{{{\text{critic}}}}$ spectrum calculated theoretically considering a range of $\alpha$ (10^–3^–10^0^ Gy^−1^) and $T_{p}$(1–350 days) parameters. The $R_{{{\text{critic}}}}$ and $R_{{{\text{critic}}\;\left( k \right)}}$ calculated using the derived clinical radiobiological parameters were shown with blue and green markers, respectively. The theoretical spectrum has shown, lesions with larger $\alpha$ and longer $T_{{\text{p}}}$ (black shaded area) has a higher $R_{{{\text{critic}}}}$. This region can represent lesions with low to medium radioresistivity. However, smaller $R_{{{\text{critic}}}}$ was predicted for lesions with smaller $\alpha$ and shorter $T_{p}$ (yellow-white shaded area). Most of the clinical $R_{{{\text{critic}}}}$ was calculated to be within black shaded area (i.e. ≈ 0.003–12 Gy/day). However, there is an evident shift towards a higher critical dose rate (the yellow-white shaded area) when the clinically driven $T_{{\text{k}}}$ was assumed to be the new $T_{{\text{p}}}$ (as it was explained in the method section, as $T_{p} \to T_{{\text{k}}}$ represents the highest acceleration in tumour proliferation rate scenario).

**Fig. 6:**
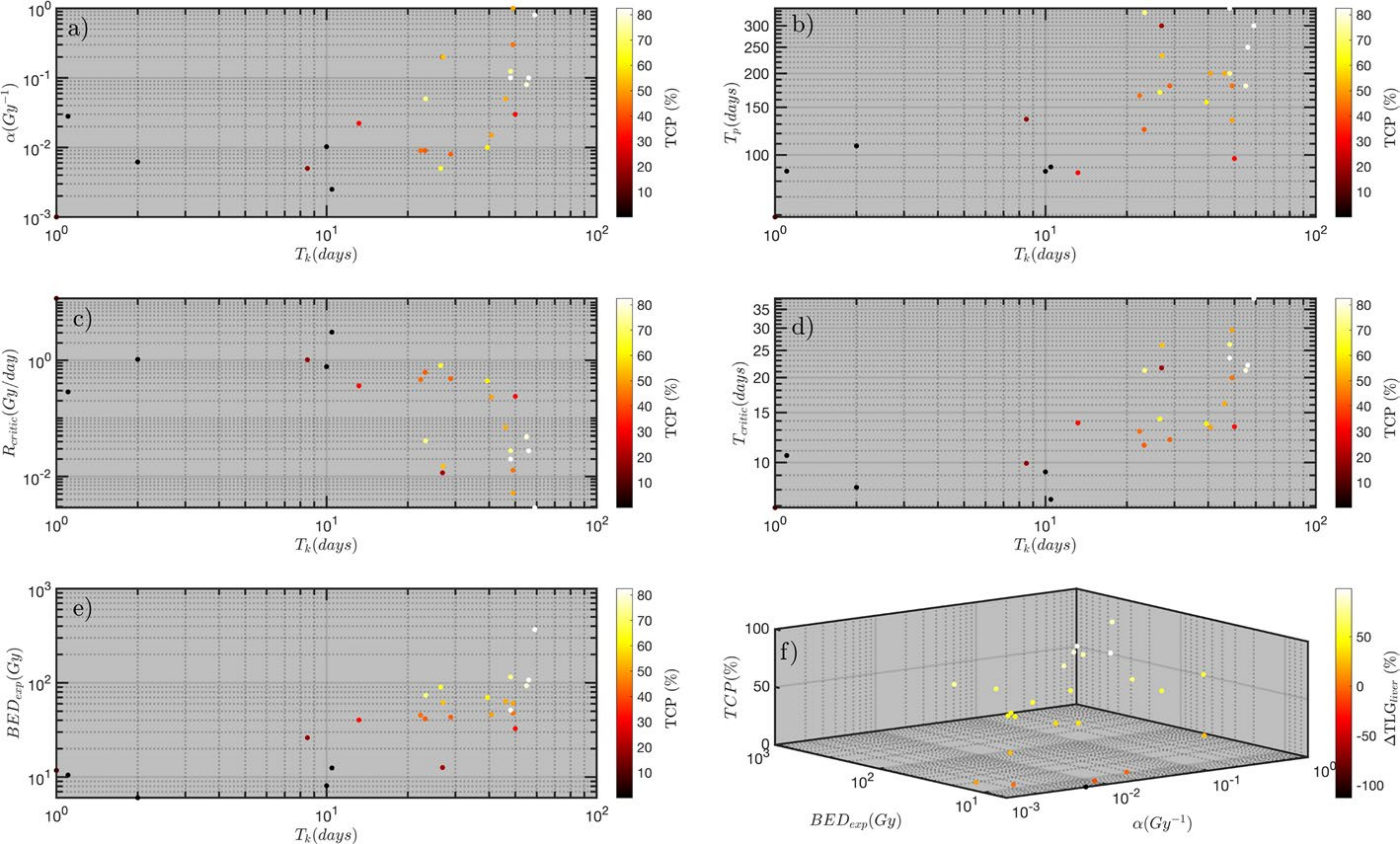
**a**–**e** The relationship between the clinically estimated radiobiological parameters and TCP. **f** 4D scatter plot demonstrating the relationship between the biological effective dose (${\text{BED}}_{{{\text{exp}}}}$), $\alpha$ parameter, TCP, and the total liver lesion glycolysis $\Delta {\text{TLG}}_{{{\text{liver}}}}$

Sub-figures in Fig. 6a–e are 3D plots which demonstrate the relationship between the clinically driven radiobiological parameters and calculated TCP. Figure 6a shows that lower and higher TCPs were achieved for radioresistant (i.e. small $\alpha$ parameter) and radiosensitive (i.e. large $\alpha$ parameter) lesions, respectively. Also, it shows that radioresistant and radiosensitive lesions tend to have a shorter and longer $T_{{\text{k}}}$, respectively. Figure 6b also shows that highly proliferating lesions (i.e. short $T_{{\text{p}}}$) have a shorter $T_{k}$ and therefore they resulted in a higher $R_{{{\text{critic}}}}$ (shown in Fig. 6c) and lower TCP. Furthermore, in Fig. 6d, e, lesions with shorter $T_{{\text{k}}}$ have shown to have the lowest $T_{{{\text{critic}}}}$ and thus lowest ${\text{BED}}_{exp}$ and TCP. Figure 6f is a 4D plot comparing the ${\text{BED}}_{{{\text{exp}}}}$, TCP and $\Delta {\text{TLG}}_{{{\text{liver}}}}$ for lesions with different $\alpha$ parameters. Lesions with smaller $\alpha$ values have shown to result in lower TCP and $\Delta {\text{TLG}}_{{{\text{liver}}}}$.

## Discussion

The value of functional images for personalised therapy and dosimetry of ^90^Y-SIRT is limited by cancer histological and genomic heterogeneity [42, 43]. This is further compounded by the limited knowledge of the clinically relevant radiobiological parameters to evaluate the biological effectiveness of the ^90^Y-SIRT. However, many clinical studies have shown that the GLQ model can demonstrate the relationship between the radiation absorbed dose to cell killing and it has achieved great success in helping researchers and clinicians to investigate response to radiation and interpret clinical outcome data, design new treatment strategies, and compare different radiation modalities [19, 23, 44–46]. The fundamental principle of the GLQ model takes account of two main cellular damages: lethal (DNA DSBs), and sub-lethal (DNA single-strand breaks, SSBs) and their repair mechanism. Furthermore, results presented in this study have shown that the GLQ model can be used to derive the clinically relevant radiobiological parameters and they can be utilised to personalise ^90^Y-SIRT dosimetry.

Four radiobiological parameters, $T_{{\text{p}}}$, $T_{{\text{k}}}$, $\alpha$, $\alpha {/}\beta$ were derived for twenty-five patients with liver malignancy. One of the main results of this analysis is that the $T_{{\text{p}}}$, or proliferation rate of tumours, is a very influential parameter affecting the BED and treatment outcome (e.g. TCP). The tumour $T_{{\text{p}}}$ was initially reported as a tool to estimate the rate of growth of pulmonary metastases from colorectal cancer [47]. Additionally, $T_{{\text{p}}}$ can be calculated based on preoperative standard diagnostic techniques and has been shown to describe the tumour biological aggressiveness of a neoplasm [48–50]. In this study it was predicted that patients with mCRC liver lesions have shortest $T_{{\text{p}}}$ with mean of ≈ 129 ± 19 days. This is within the range of $T_{{\text{p}}}$ measured in vivo (i.e. mean of ≈ 92 [51] and 150 [52]days). One previous study [51] has shown that the $T_{p}$ of hepatic metastases in patients with CRC may be a useful prognostic marker. This clinical study suggested that patients with a tumour $T_{{\text{p}}}$ of less than 92.4 days have a poor prognosis in comparison to patients who had tumour with $T_{{\text{p}}}$ of greater than 92.4 days. Furthermore, it was indicated that patients with tumours that have shorter $T_{{\text{p}}}$ carrying a greater risk of residual primary cancer in the abdominal cavity [51]. This is also consistent with the results presented in our current study. Amongst patients who had mCRC, patients with shorter $T_{{\text{p}}}$ have shown the worst TCP and metabolic response from $\Delta {\text{TLG}}_{{{\text{liver}}}}$ (e.g. $T_{{\text{p}}}$ ≈ 87 days, TCP = 0.1% and $\Delta {\text{TLG}}_{{{\text{liver}}}}$ = − 9.7%). Furthermore, the large variation in the $\alpha$ parameter for CRC patients indicates a significant variation in the type of tumour within the patients. This explains why we have also seen a larger variation in the treatment outcome for these patients. Therefore, these results suggest for an optimal and personalised treatment; it is very critical to prescribe the dose based on clinically derived radiobiological parameters.

Patients with cholangiocarcinoma have been found to have the longest $T_{{\text{p}}}$ ≈ 236 ± 62.7 days (≈ 59–336 days). This is also within the range of a previously published clinical study [53] which found a similar range for the cholangiocarcinoma tumours $T_{{\text{p}}}$ ≈ 14.5–513 days. In comparison to CRC, NET, breast, and the other cancer types, on average cholangiocarcinoma tumours are shown to have best TCP (53% ± 17%) and $\Delta {\text{TLG}}_{{{\text{liver}}}}$ (63% ± 18%) response, and they have the second shortest $T_{k}$ (28 ± 10 days). This suggests that the radiation treatment response for tumours with long $T_{{\text{p}}}$ are less influenced by the $T_{{\text{k}}}$ parameter. This is also valid for all the cancer types investigated in this study, and the general relationship between $T_{{\text{k}}}$ and $T_{{\text{p}}}$ parameters is presented in Fig. 6b. The trend of these data has shown that tumours with longer $T_{{\text{p}}}$ will have a longer $T_{{\text{k}}}$ and higher TCP.

The time factors ($T_{{\text{p}}}$, $T_{{\text{k}}}$, $T_{{{\text{critic}}}}$ and treatment time) are amongst the key elements in radiation oncology [54]. Furthermore, the importance of delays during a course of radiotherapy or relatively low dose rate RNT (compared to EBRT) with long treatment time has been investigated in recent decades, and different recommendations on the delay-compensation options have been published [24, 27]. Fast tumour cell repopulation has been suggested as the main reason why prolonging overall treatment time in EBRT or RNT reduces the therapeutic efficacy and thus the TCP and overall survival in many human tumours [55]. In our previous study we have shown that the $T_{{{\text{critic}}}}$ could be considered as another prognostic marker [23]. The $T_{{{\text{critic}}}}$ accounts for two radiobiological parameters, $T_{{\text{p}}}$ and $\alpha$ and it considers the initial dose rate for a particular treatment. Results presented in Table 2 and Fig. 5a have shown, generally, that tumours with short $T_{{\text{p}}}$ and small $\alpha$ parameters have the shortest $T_{{{\text{critic}}}}$ and thus largest $R_{{{\text{critic}}}}$. The relationship between these radiobiological parameters and quantities for all twenty-five patients is presented in Fig. 6c–d. The trend of both data showed that, regardless of cancer type, tumours with larger $T_{{\text{p}}}$ and consequently $T_{{\text{k}}}$ result in shorter $T_{{{\text{critic}}}}$ and thus larger $R_{{{\text{critic}}}}$. Moreover, as it was shown in Fig. 6e, the $T_{{\text{k}}}$ has a significant impact on the BED calculation. The short $T_{{\text{k}}}$ means that the acceleration in proliferation rate in tumours with shorter $T_{{\text{k}}}$ occurs at an early time point after the start of the treatment. As shown in Fig. 6d, this will result in further shortening the $T_{{{\text{critic}}}}$ and thus reducing the therapeutic efficacy and the TCP. Therefore, these results suggest for such tumours, the ^90^Y-SIRT will be only effective at higher initial dose rate (e.g. $R_{0}$ > 50 Gy/day which corresponds to the yellow-white shaded area in Fig. 5a; regions where $T_{{{\text{critic}}}}$ is estimated to be greater than 20 days).

Furthermore, it was estimated that the values for $\alpha$ and $\alpha {/}\beta$ parameters range in ≈ 0.001–1 Gy^−1^ and ≈ 1–49 Gy, respectively. The average $\alpha$ parameters for breast, NET, CRC and cholangiocarcinoma were, 0.045 ± 0.036 Gy^−1^, 0.331 ± 0.183 Gy^−1^, 0.010 ± 0.005 Gy^−1^ and 0.040 ± 0.022 Gy^−1^ respectively. These values are within the range of the previously published clinical data 0.054 Gy^−1^, 0.015 Gy^−1^, 0.037 Gy^−1^ clinically driven for breast, CRC, and cholangiocarcinoma cancer types [16]. To the best of our knowledge, there are no published values for clinically driven $\alpha$ parameter for NET cancer cells. These results show that patients with metastatic CRC tumours have a consistently smaller $\alpha$ parameter and are the most radioresistant tumour type amongst all the other cancer types investigated in this study. As it was shown in Fig. 6a, in general tumours with smaller $\alpha$ parameters tend to have shorter $T_{{\text{k}}}$, therefore resulting in poor TCP.

The calculated $\Delta {\text{TLG}}_{{{\text{liver}}}}$ has shown a good correlation with TCP values. The average $\Delta {\text{TLG}}_{{{\text{liver}}}}$ and TCP calculated for all twenty-five patients were, ≈ 45% ± 9% and 43 ± 6%, respectively. As it was demonstrated in Fig. 6f, tumours with larger $\alpha$ parameters have consistently resulted in high ${\text{BED}}_{{{\text{exp}}}}$, TCP and $\Delta {\text{TLG}}_{{{\text{liver}}}}$. These results suggest that $\Delta {\text{TLG}}_{{{\text{liver}}}}$ and TCP have a similar relationship with the driven radiobiological parameters and thus $\Delta {\text{TLG}}_{{{\text{liver}}}}$ could be considered as an alternative radiobiological metric to the TCP.

Finally, results from this study suggest that the standard BED formulism (i.e. ${\text{BED}}_{\infty }$) without considering the $T_{{\text{p}}}$ and $T_{{\text{k}}}$ time parameters can be a misleading metric to assess the tumour radiation biological response. For example, considering the patient case presented in Fig. 4, although both liver lesions in the left and right lobes received similar average absorbed dose of ≈ 30 Gy and ${\text{BED}}_{\infty }$≈ 32 Gy, due to shorter $T_{{\text{p}}}$ and $T_{{\text{k}}}$(≈ 92 and 23 days), the lesion in the left lobe (*L*1) resulted in a much lower TCP (i.e. ≈ 43% less than *L*2 TCP). However, when the $T_{{\text{p}}}$ and $T_{{\text{k}}}$ time parameters were included in the BED calculation (i.e. ${\text{BED}}_{{{\text{exp}}}}$), while a similar ${\text{BED}}_{\infty }$ and ${\text{BED}}_{exp}$ values (≈ 31 and 33 Gy) was achieved for *L*2, the *L*1 ${\text{BED}}_{\infty }$(≈ 32 Gy) was reduced to ${\text{BED}}_{{{\text{exp}}}}$ ≈ 22 Gy. Therefore, a better correlation between the BED and TCP was achieved.

In summary, this study demonstrates a novel approach whereby using pre- and post-treatment FDG PET/CT images, in addition to radiosensitivity of the tumour cells, valuable information on the temporal changes (such tumour $T_{{\text{k}}}$ and $T_{{\text{p}}}$) in FDG uptake of tumour could be estimated. These in vivo-driven radiobiological parameters may not only lead towards personalised dosimetry in patients with liver malignancy treated with ^90^Y-SIRT but for other targeted radionuclide therapy modalities such as Lutetium-177 therapy.

## Conclusion

Results presented in this study show that ^90^Y PET/CT with pre- and post-treatment FDG PET/CT images can be used to derive the clinically relevant radiobiological parameters (i.e. $T_{{\text{p}}}$, $T_{{\text{k}}}$, $\alpha$, $\alpha {/}\beta$) of the GLQ model. It was estimated that the values for $\alpha$ and $\alpha {/}\beta$ parameters range in ≈ 0.001–1 Gy^−1^ and ≈ 1–49 Gy respectively. We have demonstrated that the time factors, $T_{{\text{p}}}$, $T_{{\text{k}}}$, $T_{{{\text{critic}}}}$ are the key parameters when evaluating liver malignancy lesion response to [^90^Y] SIR-Spheres treatment. Patients with cholangiocarcinoma have been shown to have the longest average $T_{{\text{p}}}$ (≈ 236 ± 67 d), highest TCP (≈ 53 ± 17%) and $\Delta {\text{TLG}}_{{{\text{liver}}}}$ ≈ 64% while patients with metastatic CRC tumours have the shortest average $T_{{\text{p}}}$ (≈ 129 d ± 19 d), lowest TCP (≈ 28% ± 13%) and $\Delta {\text{TLG}}_{{{\text{liver}}}}$ ≈ 8% respectively. Therefore, these results suggest for such tumours, the ^90^Y-SIRT will be only effective at higher initial dose rate (*e.g.*  > 50 Gy/day).

## Data Availability

Data are available upon reasonable request subject to ethical approval.

## Abbreviations

BED: Biological effective dose
CMR: Complete metabolic response
COV: Coefficient of variation
CRC: Colorectal cancer
CT: Computed tomography
DVH: Dose volume histogram
DSB: DNA double-strand break
EBRT: External beam radiotherapy
GLQ: Generalised linear–quadratic
HCC: Hepatocellular carcinoma
*L*: Lesion
mCRC: Metastatic colorectal cancer
NEC: Neuroendocrine carcinoma
PDAC: Pancreatic ductal adenocarcinoma
PET: Positron emission tomography
PMD: Progressive metabolic disease
PNET: Pancreatic neuroendocrine tumours
RNT: Radionuclide therapy
SBNET: Small bowel neuroendocrine tumours
SF: Survival fraction
SSB: DNA single-strand break
SUV: Standardised uptake value
SIRT: Selective internal radionuclide therapy
SPECT: Single-photon emission computed tomography
TLG: Total liver lesion glycolysis
TCP: Tumour control probability
VSF: Voxel survival fraction
